# Copeptin levels are associated with organ dysfunction and death in the intensive care unit after out-of-hospital cardiac arrest

**DOI:** 10.1186/s13054-015-0831-y

**Published:** 2015-03-31

**Authors:** Giuseppe Ristagno, Roberto Latini, Mario Plebani, Martina Zaninotto, Jukka Vaahersalo, Serge Masson, Marjaana Tiainen, Jouni Kurola, Flavio Gaspari, Valentina Milani, Ville Pettilä, Markus Benedikt Skrifvars, for the FINNRESUSCI Study Group

**Affiliations:** Department of Cardiovascular Research, IRCCS-Istituto di Ricerche Farmacologiche Mario Negri, Via La Masa, 19 - 20156 Milano, Milan, Italy; Department of Laboratory Medicine, University-Hospital of Padova, Via Giustiniani 2, 35128 Padova, Italy; Division of Intensive Care Medicine, Department of Anaesthesiology, Intensive Care and Pain Medicine, University of Helsinki and Helsinki University Hospital, Haartmaninkatu 4, 00290 Helsinki, Finland; Department of Neurology, Helsinki University Hospital, Haartmaninkatu 4, 00290 Helsinki, Finland; Centre for Prehospital Emergency Care, Kuopio University Hospital, P.O. Box 100, FI 70029 Kuopio, Finland; Laboratory of Pharmacokinetics and Clinical Chemistry, IRCCS-Istituto di Ricerche Farmacologiche ‘Mario Negri’, Villa Camozzi, 24020 Ranica, Italy

## Abstract

**Introduction:**

We studied associations of the stress hormones copeptin and cortisol with outcome and organ dysfunction after out-of-hospital cardiac arrest (OHCA).

**Methods:**

Plasma was obtained after consent from next of kin in the FINNRESUSCI study conducted in 21 Finnish intensive care units (ICUs) between 2010 and 2011. We measured plasma copeptin (pmol/L) and free cortisol (nmol/L) on ICU admission (245 patients) and at 48 hours (additional 33 patients). Organ dysfunction was categorised with 24-hour Sequential Organ Failure Assessment (SOFA) scores. Twelve-month neurological outcome (available in 276 patients) was classified with cerebral performance categories (CPC) and dichotomised into good (CPC 1 or 2) or poor (CPC 3 to 5). Data are presented as medians and interquartile ranges (IQRs). A Mann–Whitney *U* test, multiple linear and logistic regression tests with odds ratios (ORs) 95% confidence intervals (CIs) and beta (B) values, repeated measure analysis of variance, and receiver operating characteristic curves with area under the curve (AUC) were performed.

**Results:**

Patients with a poor 12-month outcome had higher levels of admission copeptin (89, IQR 41 to 193 versus 51, IQR 29 to 111 pmol/L, *P* = 0.0014) and cortisol (728, IQR 522 to 1,017 versus 576, IQR 355 to 850 nmol/L, *P* = 0.0013). Copeptin levels fell between admission and 48 hours (*P* <0.001), independently of outcome (*P* = 0.847). Cortisol levels did not change between admission and 48 hours (*P* = 0.313), independently of outcome (*P* = 0.221). The AUC for predicting long-term outcome was weak for copeptin (0.62, 95% CI 0.55 to 0.69) and cortisol (0.62, 95% CI 0.54 to 0.69). With logistic regression, admission copeptin (standard deviation (SD) increase OR 1.4, 95% CI 1.03 to 1.98) and cortisol (SD increase OR 1.5, 95% CI 1.1 to 2.0) predicted ICU mortality but not 12-month outcome. Admission factors correlating with SOFA were shockable rhythm (B −1.3, 95% CI −2.2 to −0.5), adrenaline use (B 1.1, 95% CI 0.2 to 2.0), therapeutic hypothermia (B 1.3 95% CI 0.4-2.2), and copeptin (B 0.04, 95% CI 0.02 to 0.07).

**Conclusions:**

Admission copeptin and free cortisol were not of prognostic value regarding 12-month neurological outcome after OHCA. Higher admission copeptin and cortisol were associated with ICU death, and copeptin predicted subsequent organ dysfunction.

**Electronic supplementary material:**

The online version of this article (doi:10.1186/s13054-015-0831-y) contains supplementary material, which is available to authorized users.

## Introduction

Cardiac arrest is a devastating condition associated with high mortality and morbidity [1]. After the return of spontaneous circulation (ROSC), a pathophysiological state recently named ‘post-cardiac arrest syndrome’ is frequently observed, including myocardial dysfunction with circulatory shock and evolving brain injury [2,3]. In all types of critical illness, a stress response, accompanied by activation of the hypothalamic-pituitary-adrenal (HPA) axis and release of the hormone arginine-vasopressin (AVP), is seen [4,5]. Activation of the HPA axis results in the secretion of the adrenocorticotropic hormone (ACTH), which stimulates the medulla to synthesise cortisol [6]. Relative adrenal insufficiency is common following cardiac arrest and seems to be related to the severity of the ischaemic insult [7].

Copeptin is the C-terminal part of the AVP precursor pro-AVP [8]. Copeptin is stable and fairly easy to measure as opposed to AVP. Studies have shown that copeptin is an accurate marker of both disease severity and survival in various conditions, such as myocardial infarction, heart failure, and stroke [9-11]. Two small pilot trials have indicated excellent prognostic accuracy of copeptin in patients admitted to the intensive care unit (ICU) following out-of-hospital cardiac arrest (OHCA) [12,13]. Accordingly, the aim of the present study was to evaluate the accuracy of circulating copeptin levels at admission and 48 hours later in the prediction of long-term neurological outcomes after OHCA. For comparison, we studied free cortisol as a marker of HPA axis activation. We further sought to study the associations with admission levels of copeptin and cortisol and the severity of subsequent organ dysfunction.

## Methods

### Patient group

The patients included in this study were part of the FINNRESUSCI study, which was a prospective observational cohort study that was conducted at 21 hospitals in Finland between 1 March 2010 and 28 February 2011 and that included 548 patients [14]. The study was approved by the ethics committee of the Helsinki and Uusimaa Hospital district (FINNRESUSCI TUTKIMUS §10, 20.1.201) in addition to local ethics approvals in six of the hospitals (listed in the Appendix). Informed consent was obtained from the next of kin prior to obtaining blood samples. Informed consent was obtained for 245 patients at the time of ICU admission and for an additional 33 patients prior to additional sampling 48 hours later, giving a total study sample of 278 patients. Information on the 12-month neurological outcome was available for 276 patients. Thus, these 276 patients were included in the long-term outcome analysis.

### Assay of circulating stress markers

Blood samples were collected in ethylenediaminetetraacetic acid tubes and centrifuged, and the plasma was stored at −70°C. Upon analysis, the samples were thawed and divided into aliquots. The plasma concentration of cortisol was measured by a chemiluminescent immunoassay on an Access 2 instrument (Beckman Coulter S.p.A., Cassina De’ Pecchi, Italy). The imprecision of the assay was less than 10%. The reference ranges for plasma cortisol were 185 to 624 nmol/L (morning) and less than 276 nmol/L (post-meridiem). The concentrations of copeptin were assayed by using an immunofluorescent immunoassay (Copeptin; Brahms, Henningsdorf, Germany) with an automated Kriptor Analyzer (Thermo Fisher Scientific, Milan, Italy). The limit of detection was 0.9 pmol/L, and the lower concentration measurable with a coefficient of variation of less than 10% was less than 4 pmol/L. The 95th percentile calculated in healthy donors was less than 12 pmol/L. All of the assays were performed in a centralised laboratory by personnel blinded to the clinical characteristics and patient outcome.

### Data collection

All the participating hospitals are part of the Finnish Intensive Care Consortium, and all the ICUs, except one, use the same electronic data management systems and data validation software (Web Validator; Tieto, Helsinki, Finland). Patient data were collected prospectively by using internet-based case report forms. Pre-hospital data were collected by paramedics in accordance with the Utstein guidelines and included factors such as whether the arrest was witnessed, the administration of bystander-initiated life support, time from the call to the dispatch centre and the ROSC, and the use of adrenaline [15]. In-hospital care data were collected electronically and included the Simplified Acute Physiology Score II (SAPS II) and ICU and hospital mortality. The Sequential Organ Failure Assessment (SOFA) score was used as a measure of organ function during the first 24 hours of ICU care.

### Survival and 12-month neurological outcomes

The time of death was recorded for each patient. A specialist in neurology (MT) who was blinded to the ICU care and blood test results evaluated the patient’s neurological outcome according to the Pittsburgh Cerebral Performance Category (CPC) [15]. A good outcome was defined as a CPC of 1 or 2, and a poor outcome was classified as a CPC of 3 to 5. Patients or their next of kin were contacted by phone, and the outcome was determined in a structured fashion. If a patient was hospitalized or in a nursing home, nursing staff or relatives were contacted by phone.

### Statistical analysis

Descriptive statistics are expressed with counts and percentages for categorical variables and median values with the interquartile range (IQR) for continuous variables. The levels of plasma copeptin and free cortisol on admission were compared with the Mann–Whitney *U* test, and changes over time between admission and 48 hours later were assessed with a repeated measures analysis of variance. We used the appropriate correction for a non-normal distribution, if needed, and analysed the differences between those with a good and a poor outcome and the interaction with time. Correlations between admission factors and subsequent 24-hour SOFA scores were analysed with univariate and multiple linear regression with corresponding beta values and 95% confidence intervals (CIs). Multi-variable logistic regression was used to identify factors at admission that were independent predictors of ICU mortality and a good outcome at 12 months. All of the variables associated with the outcome in the univariate analysis (*P* <0.05) and factors found to be relevant in previous OHCA studies were included in the multiple linear and logistic regression models. The prognostic discrimination of copeptin and cortisol for 12-month outcomes was investigated by using receiver operating characteristic (ROC) curves, with the corresponding area under the curve (AUC). We compared the AUCs by using a bootstrap method (2,000 samples) [16]. Odds ratios (ORs) with their corresponding 95% CIs were calculated, and *P* values were considered statistically significant if they were less than 0.05. All statistical analyses were performed with SAS software, version 9.2 (SAS Institute, Inc., Cary, NC, USA), SPSS version 19.0 (SPSS Inc., Chicago, IL, USA), and GraphPad Prism 6.0 (GraphPad Software, Inc., La Jolla, CA, USA).

## Results

Of the included 278 patients, 183 (66%) had a cardiac arrest with an initial shockable rhythm, and 95 (34%) had a non-shockable rhythm. The majority of the patients (n = 202, 73%) were treated with therapeutic hypothermia (TH), and 76 (27%) were not. Of the 278 patients, 229 (82%) survived to ICU discharge, 170 (59%) survived to hospital discharge, and 143 (52%) of the patients were alive at 12 months. Of the 143 patients who survived 12 months, 133 (48% of the total 276 resuscitated patients) had a favourable neurological outcome (CPC of 1 or 2). Long-term data were not available in two patients.

Among the 183 patients with a shockable rhythm, 166 (91%) survived to ICU discharge, and 110 (60%) had a good neurological outcome at 12 months. Among the 95 patients with a non-shockable rhythm, 63 (69%) survived to ICU discharge, and 23 (24%) had a good 12-month neurological outcome (long-term outcome data missing for one patient). Several factors at resuscitation were associated with a higher likelihood of ICU survival. These included a shockable initial rhythm, no use of adrenaline, a shorter delay to ROSC, and treatment with TH (Table 1). Factors correlated with a good 12-month outcome were younger age, a shockable rhythm, witnessed arrest, no use of adrenaline, and a shorter time to ROSC (Table 1). The results of the multiple logistic regression models are shown in Tables 2 and 3.

**Table 1:** **Baseline characteristics and copeptin and cortisol levels of out-of-hospital cardiac arrest patients and short-term and long-term outcomes**

	**ICU non-survivors (n = 49)**	**ICU survivors (n = 229)**	***P*** **value**	**Poor 12-month outcome (n = 143)**	**Good 12-month outcome (n = 133)**	***P*** **value**
Patient factors						
Age, years	64±14	63±12	0.191	65 (±13)	60 (±12)	0.0017
Male,% (n)	82% (40)	83% (189)	0.881	83% (119)	82% (109)	0.782
Factors at resuscitation						
Shockable,% (n)	35% (17)	71 (163)	<0.001	50% (71)	81% (108)	<0.001
Time to ROSC, minutes	24 (20–31)	18 (12–26)	<0.001	23 (18-30)	16 (11-23)	<0.001
Adrenaline used, yes	92% (45)	62% (141)	<0.001	83% (118)	50% (67)	<0.001
ICU treatment						
Therapeutic hypothermia,% (n)	59% (29)	76% (173)	0.020	68% (97)	78% (104)	0.053
Lowest 24-hour MAP, mm Hg	58 (41–64)	62 (58–68)	<0.001	61 (55–67)	61 (57–68)	0.241
Highest 24-hour norepinephrine, μg/kg per minute	0.18 (0–0.35)	0.06 (0–0.13)	<0.001	0.07(0–0.19)	0.05 (0–0.14)	0.409
SAPS II	73 (64–81)	57 (39–66)	<0.001	66 (57–73)	48 (34–63)	<0.001
Biomarkers						
Copeptin 0–6 hours, pmol/L	148 (58–291)	63 (32–131)	0.0009	89 (41–193)	51 (29–111)	0.0014
Copeptin 48 hours, pmol/L	20 (9–111)	20 (11–40)	0.3399	23 (13–44)	16 (10–46)	0.0702
Relative change in copeptin	−45 (−166-(−13))	−38 (−100-(−11))	0.502	−44 (−142-(−13))	−32 (−88-(7))	0.152
Cortisol 0–6 hours, nmol/L	860 (627–1,119)	634 (378–876)	0.0006	728 (522–1,017)	576 (355–850)	0.0013
Cortisol 48 hours, nmol/L	627 (311–1,461)	548 (378–813)	0.4414	645 (403–942)	480 (328–733)	0.0053
Change in cortisol,%	−115 (−592-253)	−62 (−329-192)	0.419	−59 (−343-215)	−65 (−331-194)	0.741

One hundred eighty-nine patients were included in the delta copeptin/cortisol analysis of 12-month outcomes, and 190 patients were included in the delta copeptin/cortisol analysis of intensive care unit (ICU) mortality. MAP, mean arterial pressure; ROSC, return of spontaneous circulation; SAPS II, Simplified Acute Physiology Score II.

**Table 2:** **Results of multi-variable regression analysis of factors associated with a poor long-term neurological outcome**

**Variables**	**Risk category**	**Model 1 (with cortisol)**	***P*** **value**	**Model 2 (with copeptin)**	***P*** **value**
		**OR (95% CI)**		**OR (95% CI)**	
Age	Increase/year	1.05 (1.03-1.08)	<0.001	1.05 (1.02-1.08)	<0.001
Witnessed arrest	Yes	0.5 (0.15-1.5)	0.210	0.5 (0.14-1.5)	0.194
Initial rhythm	Not shockable	4.5 (2.2-9.3)	<0.001	4.5 (2.2-9.3)	<0.001
Time to ROSC	Increase/minute	1.08 (1.04-1.12)	<0.001	1.07 (1.03-1.12)	<0.001
Adrenaline	Yes	2.5 (1.2-5.2)	0.019	2.4 (1.12-5.1)	0.024
Therapeutic hypothermia	Yes	0.7 (0.3-1.5)	0.321	0.7 (0.3-1.5)	0.33
Cortisol	Increase/SD	1.1 (0.8-1.5)	0.535	-	-
Copeptin	Increase/SD	-	-	1.2 (0.8-1.8)	0.340

CI, confidence interval; OR, odds ratio; ROSC, return of spontaneous circulation; SD, standard deviation.

**Table 3:** **Results of multi-variable logistic regression analysis of intensive care unit mortality**

**Variables**	**Risk category**	**Model 1 (with cortisol)**	***P*** **value**	**Model 2 (with copeptin)**	***P*** **value**
		**OR (95% CI)**		**OR (95% CI)**	
Initial rhythm	Not shockable	3.9 (1.7-8.6)	0.0010	3.8 (1.7-8.5)	<0.001
Time to ROSC	Increase/minute	1.03 (0.99-1.07)	0.111	1.03 (0.99-1.06)	0.326
Use of adrenaline	Yes	5.3 (1.5-19.1)	0.010	4.1 (1.3-13.3)	0.020
Therapeutic hypothermia	Yes	0.6 (0.3-1.4)	0.1528	0.5 (0.2-1.3)	0.199
Cortisol	Increase/SD	1.5 (1.1-2.0)	0.013	-	-
Copeptin	Increase/SD	-	-	1.4 (1.03-1.98)	0.034

CI, confidence interval; OR, odds ratio; ROSC, return of spontaneous circulation; SD, standard deviation.

### Levels of copeptin and cortisol and patient outcomes

**Figure 1:**
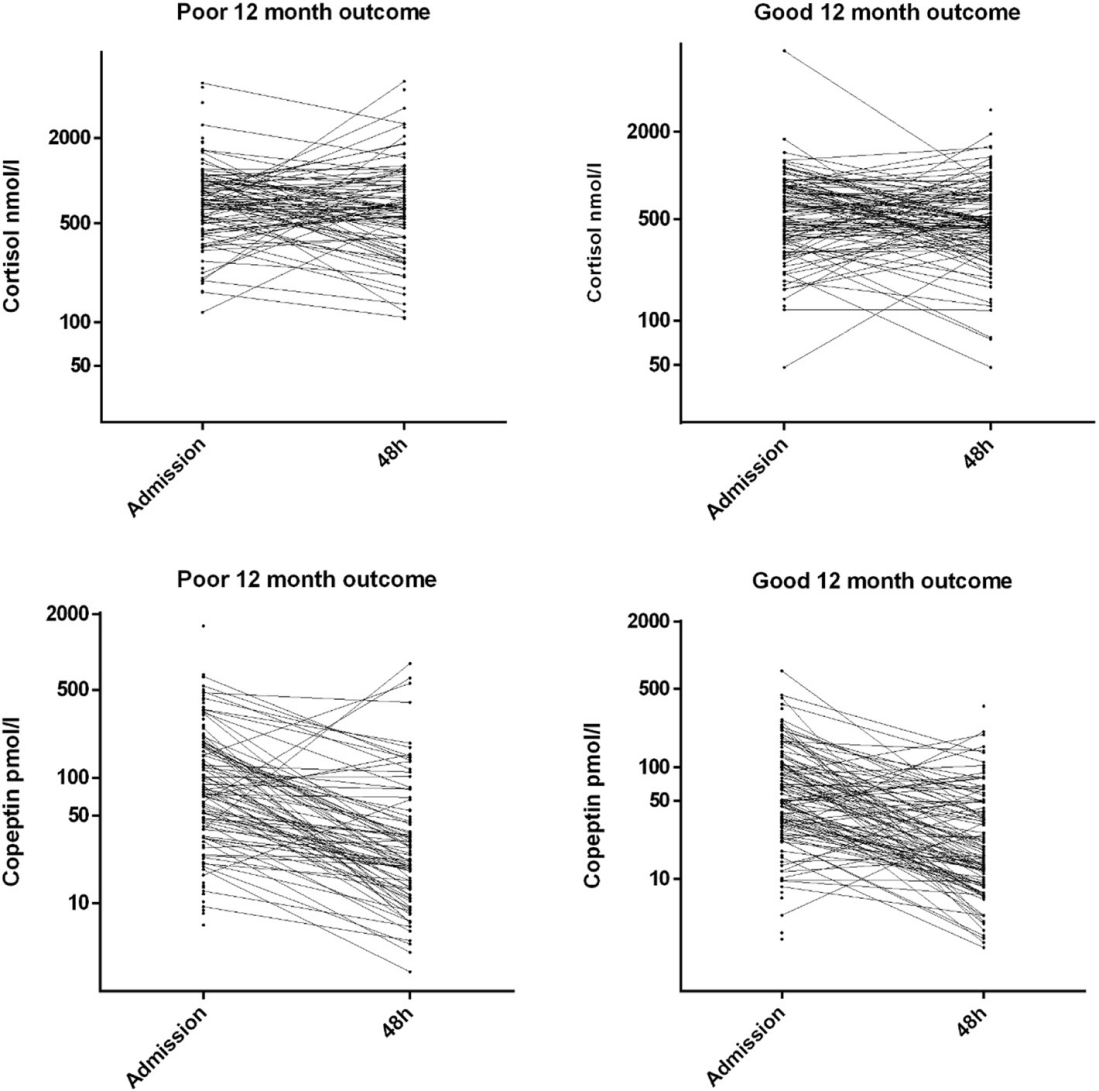
**Association of plasma copeptin and free cortisol values at the time of admission and 48 hours later with 12-month outcomes.** Copeptin levels changed over time in both groups (*P* <0.001), with no interaction between outcome and time (*P* = 0.847). There was no significant change over time (*P* = 0.313) and no interaction between time, patient outcomes (*P* = 0.221), and cortisol levels.

Median copeptin levels on admission were higher in patients who died in the ICU (148 pmol/L, IQR 58 to 291) and in those with a poor 12-month outcome (89 pmol/L, IQR 41 to 193) compared with those who survived to ICU discharge (63 pmol/L, IQR 32 to 131) and those with a good 12-month outcome (51 pmol/L, IQR 29 to 111) (*P* = 0.0009 and *P* = 0.0014). The differences in the levels of copeptin measured at 48 hours were not significant (Table 1). The levels of copeptin changed over time in both groups (*P* <0.001), but the interaction between patient outcomes and time was not significant (*P* = 0.847) (Figure 1). In the multi-variate analysis, copeptin was an independent predictor of ICU survival but not 12-month outcomes (Tables 2 and 3). The AUCs were 0.623 (0.553 to 0.693) for a poor 12-month outcome and 0.656 (0.558 to 0.754) for ICU mortality (Additional file 1). When only the patients treated with TH were analysed, the corresponding values were 0.63 (0.54 to 0.71) for long-term outcome and 0.71 (0.59 to 0.83) for ICU mortality.

Median admission cortisol levels were higher in those who died in the ICU (860 nmol/L, 627 to 1,119) and in those with a poor outcome at 12 months (728 (522 to 1017), IQR 403 to 942) compared with those who survived to ICU discharge (634 nmol/L, 378 to 876) and those with a good 12-month outcome (576 (355 to 850), IQR 328 to 733). There was no significant change over time (*P* = 0.313) and no interaction between time and outcome (*P* = 0.221) (Figure 1). In the multi-variate analysis, cortisol was an independent predictor of ICU death but not of 12-month outcomes (Tables 2 and 3). The AUCs were 0.624 (0.554 to 0.694) for a poor 12-month outcome and 0.662 (0.574 to 0.750) for ICU mortality (Additional file 1). When only the TH patients were analysed, the corresponding figures were 0.58 (0.50 to 0.66) for long-term outcomes and 0.61 (0.5 to 0.73) for ICU mortality. There was no difference in the AUCs of copeptin and cortisol for ICU mortality (*P* = 0.91) or for poor 12-month mortality prediction (*P* = 0.97).

### Copeptin, cortisol, and organ failure

**Figure 2:**
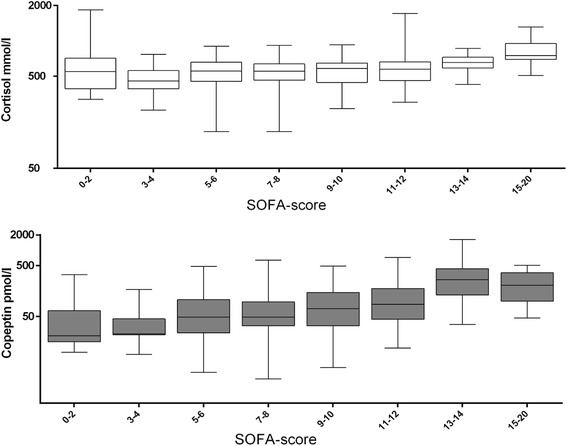
**Plasma-free cortisol and copeptin values measured on admission and subsequent 24-hour Sequential Organ Failure Assessment (SOFA) scores.** Copeptin, but not cortisol, was an independent predictor of SOFA score in a multiple linear regression.

Admission copeptin and cortisol levels indexed by the severity of the subsequent SOFA score are shown in Figure 2. Factors correlated with increased severity of organ failure in the univariate analysis were a non-shockable initial rhythm, use of adrenaline, prolonged time to ROSC, and admission copeptin and cortisol levels (Table 4). In the multiple linear regression, only a non-shockable rhythm, adrenaline, use of TH, and admission copeptin levels were independent predictors of a higher SOFA score (Table 4).

**Table 4:** **Factors associated with the first 24-hour Sequential Organ Failure Assessment scores**

**Factors**		**Univariable analysis**		**Multi-variable analysis**	
	**Risk category**	**Β (95% CI)**	***P*** **value**	**Β (95% CI)**	***P*** **value**
Age	Per 10-year increase	0.193 (−0.1-0.486)	0.195	0.02 (−0.08-0.47)	0.158
Gender	Male	0.351 (−0.634-1.337)	0.484	NI	NI
Shockable initial rhythm	Yes	−1.530 (−2.301-0.758)	<0.001	−1.34 (−2.16-(−0.51))	0.002
Time to ROSC	Per 10-minute increase	0.768 (0.437-1.099)	<0.001	0.193 (−0.20-0.59)	0.338
Adrenaline	Yes	1.939 (1.174-2.704)	<0.001	1.1 (0.22-1.99)	0.014
Bystander CPR	Yes	−0.126 (−0.884-0.633)	0.745	NI	NI
Witnessed arrest	Yes	−0.498 (−1.835-0.839)	0.464	NI	NI
Therapeutic hypothermia	Yes	0.820 (−0.018-1.658)	0.055	1.27 (0.36-2.17)	0.006
Copeptin	Per increase of 10 pmol/L	0.06 (0.038-0.082)	<0.001	0.043 (0.02-0.066)	<0.001
Cortisol	Per increase of 100 nmol/L	0.047 (0.001-0.093)	0.046	0.03 (−0.014-0.074)	0.179

CI, confidence interval; CPR, cardiopulmonary resuscitation; NI, not included; ROSC, return of spontaneous circulation.

## Discussion

In this multi-centre study, we found that plasma copeptin and free cortisol levels were markedly increased in OHCA patients at the time of admission to the ICU. The level of copeptin, but not that of free cortisol, normalised at 48 hours independently of the patient outcome. Plasma copeptin was an independent predictor of organ dysfunction and ICU death but not of long-term outcome. Copeptin, but not free cortisol, levels on admission correlated independently with the severity of organ dysfunction.

Two previous small pilot studies on OHCA outcomes and plasma copeptin levels were limited to patients treated with TH, and these found that the predictive value of copeptin was good [12,13]. In a study of 40 patients, Ostadal *et al*. showed that copeptin had an AUC of 0.801 for the prediction of 30-day mortality [12]. They reported that a copeptin level exceeding a cutoff value of 217.9 pmol/L was significantly associated with increased mortality at 30 days. Annborn *et al*. presented comparable findings in a study including 80 cardiac arrest patients treated with TH [13]. In that study, copeptin measured at 12 hours had the best predictive power, with an AUC of 0.85. Interestingly, in that study, there was less overlap between patients with good and poor outcomes 12 and 24 hours after cardiac arrest. It is conceivable that the sampling times that we used in this study were outside the optimal time interval for prognostication using copeptin.

Organ dysfunction is common after cardiac arrest and is correlated with survival [17]. Levels of inflammatory markers, especially those of interleukin-6, are associated with the severity of organ dysfunction in OHCA patients treated with targeted temperature management [18,19]. The activation of the HPA axis is a crucial part of the endocrine stress response, and several factors activate the HPA axis. Interestingly, a recent study demonstrated that global hypoxia stimulated copeptin release in an experimental setting [20]. Upon exposure to hypoxia, copeptin levels increased but returned to near normal levels 16 hours later, despite continued exposure to hypoxia [20]. This is in line with the time pattern of copeptin levels seen in the present study. Newborns have high copeptin levels [21]. In patients with myocardial infarction, copeptin levels are higher in those with acute heart failure [22]. Cardiac arrest patients have both severe hypotension and global hypoxia, both of which may stimulate the release of AVP. The release of AVP is further stimulated by inflammation and the release of inflammatory markers intereukin-1 and interleukin-6 [23]. Taken together, our findings suggest that copeptin release is induced mainly by the ischaemic insult in cardiac arrest and that it may play a role in the evolution of organ dysfunction after cardiac arrest.

In the present study, cortisol levels were elevated and remained so at 48 hours after admission to the ICU, corroborating the findings of previous studies of cortisol levels in patients with OHCA. Not only the release of ACTH but also adrenal function influences cortisol levels. Thus, low levels of free cortisol may suggest adrenal failure and may be a sign of a poor prognosis in patients with OHCA, especially in response to increased levels of ACTH [23,24]. The evidence for the value of basal cortisol levels on admission after OHCA has been conflicting; some studies indicate lower levels [7] and others report higher levels [24,25] in survivors. In the present study, plasma cortisol levels remained greatly elevated, even at 48 hours, in those with good and those with poor outcomes.

### Study strengths and limitations

The strengths of the present study include the study size and multi-centre setting. First, the demographic data of the study population suggest that our patient sample is representative of the whole FINNRESUSCI OHCA population and therefore has external validity. Second, the primary outcome, 12-month CPC, was prospectively determined by a researcher (MT) blinded to biomarker levels and intensive care management. Nonetheless, this study is not without limitations. First, owing to the ethical requirement for informed consent prior to blood sampling, not all patients could be included and there was a variation in time from cardiac arrest to sampling. Second, we measured plasma-free cortisol levels only. We did not perform ACTH tests, as these are not recommended for critically ill patients.

## Conclusions

Higher plasma copeptin and free cortisol levels on admission after OHCA predicted ICU mortality but not long-term neurological outcomes after OHCA, as reported earlier in small cohort studies. The level of copeptin on admission was associated with subsequent organ dysfunction. Thus, it may have clinical value for early triage decisions in patients at risk of developing organ dysfunction associated with post-cardiac arrest syndrome.

### Ethics

The ethics committees of Päijät-Häme, Etelä-Karala, Satakunta, and Kymenlaakso Central Hospitals and Tampere and Turku University Central Hospitals in addition to the Helsinki and Uusimaa Hospital District approved the study.

## Key messages

- Copeptin measured at the time of ICU admission is elevated but decreases to near-normal levels 48 hours later, whereas cortisol remains elevated for 48 hours after OHCA.
- Copeptin is an accurate marker of ICU death and the severity of organ dysfunction but is of limited value in isolation for long-term neurological outcomes.

## Appendix

### The FINNRESUSCI Study Group

Satakunta Central Hospital—Vesa Lund Päivi Tuominen, Satu Johansson, Pauliina Perkola, and Elina Kumpulainen; East Savo Central Hospital—Markku Suvela Sari Hirvonen, and Sirpa Kauppinen; Central Finland Central Hospital—Raili Laru-Sompa and Mikko Reilama; South Savo Central Hospital—Heikki Laine Pekka Kettunen, and Iina Smolander; North Karelia Central Hospital—Matti Reinikainen and Tero Surakka; Seinäjoki Central Hospital—Kari Saarinen Pauliina Lähdeaho and Johanna Soini; South Carelia Central Hospital—Seppo Hovilehto Päijät-Häme; Central Hospital—Pekka Loisa Alli Parviainen and Pirjo Tuomi Vaasa; Central Hospital—Simo-Pekka Koivisto and Raku Hautamäki; Kanta-Häme Central Hospital—Ari Alaspää and Tarja Heikkilä; Lappi Central Hospital—Outi Kiviniemi and Esa Lintula; Keski-Pohjanmaa Central Hospital—Tadeusz Kaminski and Jane Roiko Kymenlaakso; Central Hospital—Seija Alila, Jussi Pentti, and Reija Koskinen; Länsi-Pohja’s Central Hospital—Jorma Heikkinen; Helsinki University Hospital, Jorvi Hospital— Jukka Vaahersalo, Tuomas Oksanen, Tero Varpula Anne Eronen, Teemu Hult, and Taina Nieminen; Meilahti Hospital Medical ICU—Tom Bäcklund and Leevi Kauhanen; Meilahti Hospital ICU—Kirsi-Maija Kaukonen, Ville Pettilä Leena Pettilä, and Sari Sutinen; Turku University Hospital—Juha Perttilä and Keijo Leivo; Tampere University Hospital—Sanna Hoppu, Jyrki Tenhunen, Sari Karlsson, Atte Kukkurainen, Simo Varila, Samuli Kortelainen, and Minna-Liisa Peltola; Kuopio University Hospital—Pamela Hiltunen, Jouni Kurola, Esko Ruokonen, Elina Halonen, Saija Rissanen, and Sari Rahikainen; and Oulu University Hospital—Risto Ahola, Tero Ala-Kokko, and Sinikka Sälkiö.

## Additional file

Additional file 1:
**Receiver operating characteristic curves of the predictive value of plasma copeptin and free cortisol and intensive care unit (ICU) death and poor 12-month outcome.** There was no difference in the areas under the curve (AUCs) of copeptin and cortisol for the prediction of ICU mortality (0.91) or poor 12-month outcome (0.97).

## Abbreviations

ACTH: adrenocorticotropic hormone
AUC: area under the curve
AVP: arginine-vasopressin
CI: confidence interval
CPC: (Pittsburgh) Cerebral Performance Category
HPA: hypothalamic-pituitary-adrenal
ICU: intensive care unit
IQR: interquartile range
OHCA: out-of-hospital cardiac arrest
ROSC: return of spontaneous circulation
SOFA: Sequential Organ Failure Assessment
TH: therapeutic hypothermia

## References

[1] Sasson C, Rogers MA, Dahl J, Kellermann AL (2010). Predictors of survival from out-of-hospital cardiac arrest: a systematic review and meta-analysis. Circ Cardiovasc Qual Outcomes.

[2] Peberdy MA, Callaway CW, Neumar RW, Geocadin RG, Zimmerman JL, Donnino M (2010). Part 9: post-cardiac arrest care American Heart Association guidelines for cardiopulmonary resuscitation and emergency cardiovascular care. Circulation.

[3] Kim J, Kim K, Lee JH, Jo YH, Kim T, Rhee JE (2013). Prognostic implication of initial coagulopathy in out-of-hospital cardiac arrest. Resuscitation.

[4] Adrie C, Adib-Conquy M, Laurent I, Monchi M, Vinsonneau C, Fitting C (2002). Successful cardiopulmonary resuscitation after cardiac arrest as a ‘sepsis-like’ syndrome. Circulation.

[5] Cooper MS, Stewart PM (2003). Corticosteroid insufficiency in acutely ill patients. N Engl J Med.

[6] Pene F, Hyvernat H, Mallet V, Cariou A, Carli P, Spaulding C (2005). Prognostic value of relative adrenal insufficiency after out-of-hospital cardiac arrest. Intensive Care Med.

[7] Kim JJ, Hyun SY, Hwang SY, Jung YB, Shin JH, Lim YS (2011). Hormonal responses upon return of spontaneous circulation after cardiac arrest: a retrospective cohort study. Crit Care.

[8] Katan M, Fluri F, Morgenthaler NG, Schuetz P, Zweifel C, Bingisser R (2009). Copeptin: a novel, independent prognostic marker in patients with ischemic stroke. Ann Neurol.

[9] Potocki M, Reichlin T, Thalmann S, Zellweger C, Twerenbold R, Reiter M (2012). Diagnostic and prognostic impact of copeptin and high-sensitivity cardiac troponin T in patients with pre-existing coronary artery disease and suspected acute myocardial infarction. Heart.

[10] Masson S, Latini R, Carbonieri E, Moretti L, Rossi MG, Ciricugno S (2010). The predictive value of stable precursor fragments of vasoactive peptides in patients with chronic heart failure: data from the GISSI-heart failure (GISSI-HF) trial. Eur J Heart Fail.

[11] Miller WL, Hartman KA, Grill DE, Struck J, Bergmann A, Jaffe AS (2012). Serial measurements of midregion proANP and copeptin in ambulatory patients with heart failure: incremental prognostic value of novel biomarkers in heart failure. Heart.

[12] Ostadal P, Kruger A, Zdrahalova V, Janotka M, Vondrakova D, Neuzil P (2012). Blood levels of copeptin on admission predict outcomes in out-of-hospital cardiac arrest survivors treated with therapeutic hypothermia. Crit Care.

[13] Annborn M, Dankiewicz J, Nielsen N, Rundgren M, Smith JG, Hertel S (2014). CT-proAVP (copeptin), MR-proANP and peroxiredoxin 4 after cardiac arrest: release profiles and correlation to outcome. Acta Anaesthesiol Scand.

[14] Vaahersalo J, Hiltunen P, Tiainen M, Oksanen T, Kaukonen KM, Kurola J (2013). Therapeutic hypothermia after out-of-hospital cardiac arrest in Finnish intensive care units: the FINNRESUSCI study. Intensive Care Med.

[15] Cummins RO, Chamberlain DA, Abramson NS, Allen M, Baskett PJ, Becker L (1991). Recommended guidelines for uniform reporting of data from out-of-hospital cardiac arrest: the Utstein Style. A statement for health professionals from a task force of the American Heart Association, the European Resuscitation Council, the Heart and Stroke Foundation of Canada, and the Australian Resuscitation Council. Circulation.

[16] Robin X, Turck N, Hainard A, Tiberti N, Lisacek F, Sanchez J-S (2011). pROC: an open-source package for R and S+ to analyze and compare ROC curves. BMC Bioinformatics.

[17] Roberts BW, Kilgannon JH, Chansky ME, Mittal N, Wooden J, Parrillo JE (2013). Multiple organ dysfunction after return of spontaneous circulation in postcardiac arrest syndrome. Crit Care Med.

[18] Fries M, Stoppe C, Brücken D, Rossaint R, Kuhlen R (2009). Influence of mild therapeutic hypothermia on the inflammatory response after successful resuscitation from cardiac arrest. J Crit Care.

[19] Bro-Jeppesen J, Kjaergaard J, Wanscher M, Nielsen N, Friberg J, Bjerree M (2014). The inflammatory response after out-of-hospital cardiac arrest is not modified by targeted temperature management at 33°C or 36°C. Resuscitation.

[20] Ostergaard L, Rudiger A, Wellmann S, Gammella E, Beck-Schimmer B, Struck J (2014). Arginine-vasopressin marker copeptin is a sensitive plasma surrogate of hypoxic exposure. Hypoxia.

[21] Wellmann S, Benzing J, Cippà G, Admaty D, Creutzfeldt R, Mieth RA (2010). High copeptin concentrations in umbilical cord blood after vaginal delivery and birth acidosis. J Clin Endocrinol Metab.

[22] Voors AA, von Haehling S, Anker SD, Hillege HL, Struck J, Hartmann O (2009). C-terminal provasopressin (copeptin) is a strong prognostic marker in patients with heart failure after an acute myocardial infarction: results from the OPTIMAAL study. Eur Heart J.

[23] Judd AM, Call GB, Barney M, McIlmoil CJ, Balls AG, Adams A (2000). Possible function of IL-6 and TNF as intra-adrenal factors in the regulation of adrenal steroid secretion. Ann N Y Acad Sci.

[24] Ito T, Saitoh D, Takasu A, Kiyozumi T, Sakamoto T, Okada Y (2004). Serum cortisol as a predictive marker of the outcome in patients resuscitated after cardiopulmonary arrest. Resuscitation.

[25] Tavakoli N, Bidari A, Shams Vahdati S (2012). Serum cortisol levels as a predictor of neurologic survival in successfully resuscitated victims of cardiopulmonary arrest. J Cardiovasc Thorac Res.

