# Supplementary material for: Copeptin levels are associated with organ dysfunction and death in the intensive care unit after out-of-hospital cardiac arrest
Source: Crit Care. 2015 Mar 31;19(1):132. doi: 10.1186/s13054-015-0831-y (PMC4415235; doi:10.1186/s13054-015-0831-y)
Supplement: Additional file 1: — Receiver operating characteristic curves of the predictive value of plasma copeptin and free cortisol and intensive care unit (ICU) death and poor 12-month outcome. There was no difference in the areas under the curve (AUCs) of copeptin and cortisol for the prediction of ICU mortality (0.91) or poor 12-month outcome (0.97). [file 13054_2015_831_MOESM1_ESM.docx]

**Electronic supplementary material:**

**Figure 3 (ESM) Receiver operating characteristic curves of the predictive value of plasma copeptin and free cortisol and ICU death and poor 12 month outcome.** There was no difference in the AUCs of copeptin and cortisol for the prediction of ICU mortality (0.91) or poor 12 month outcome (0.97).
